# Hospital Mortality Associated with Stroke in Southern Iran

**Published:** 2013-12

**Authors:** Afshin Borhani-Haghighi, Rasool Safari, Seyed Taghi Heydari, Faroq Soleimani, Maryam Sharifian, Sara Yektaparast Kashkuli, Mahsa Nayebi Khayatghuchani, Mahbube Azadi, Abdolhamid Shariat, Anahid Safari, Kamran Bagheri Lankarani, Amer Alshekhlee, Salvador Cruz-Flores

**Affiliations:** 1 Health Policy Research Center, School of Medicine, Shiraz University of Medical Sciences, Shiraz, Iran;; 2 Department of Neurology, Motahari Clinic, Shiraz University of Medical Sciences, Shiraz, Iran;; 3 Student Research Committee, Shiraz University of Medical Sciences, Shiraz, Iran;; 4 Department of Biostatistics, Jahrom University of Medical Sciences, Jahrom, Iran;; 5 Transgenic Technology Research Center, Shiraz University of Medical Sciences, Shiraz, Iran;; 6 Research Center for Traditional Medicine and History of Medicine, Shiraz University of Medical Sciences, Shiraz, Iran;; 7 Department of Pharmacology, School of Medicine, Kazeroon Azad University, Kazeroon , Iran;; 8 Department of Neurology and Psychiatry , Saint Louis University, Saint Louis, USA;; 9 Department of Neurology, Texas Tech University Health Sciences Center at El Paso, Texas, USA

**Keywords:** Stroke, Cerebrovascular disease, Cerebrovascular accident, Mortality, Sex

## Abstract

**Background:** Unlike the western hemisphere, information about stroke epidemiology in southern Iran is scarce. The aim of this study was to determine the main epidemiological characteristics of patients with stroke and its mortality rate in southern Iran.

**Methods:** A retrospective, single-center, hospital-based longitudinal study was performed at Nemazee Hospital in Shiraz, Southern Iran. Patients with a diagnosis of hemorrhagic and ischemic strokes were identified based on the International Classification of Diseases, 9th and 10th editions, for the period between 2001 and 2010. Demographics including age, sex, area of residence, socioeconomic status, length of hospital stay, and discharge destinations were analyzed in association with mortality.

**Results: **16351 patients with a mean age of 63.4 years (95% CI: 63.1, 63.6) were included in this analysis. Men were slightly predominant (53.6% vs. 46.4%). Forty-seven percent of the total sample was older than 65,17% were younger than 45, and 2.6% were children younger than 18. The mean hospital stay was 6.3 days (95% CI: 6.2, 6.4). Among all types of strokes, the overall hospital mortality was 20.5%. Multiple logistic regression revealed significantly higher in-hospital mortality in women and children (P<0.001) but not in patients with low socioeconomic status or from rural areas. During the study period, the mortality proportions increased from 17.8% to 22.2%.

**Conclusion: **In comparison to western countries, a larger proportion of our patients were young adults and the mortality rate was higher.

## Introduction

There has been a significant decrease in stroke mortality rates in developed countries, but this success story has not been mirrored in developing countries.^1^ Of 5.7 million stroke patients who died in 2005, 87% were from low and middle-income countries, where stroke is considered a major disabling health problem.^2^^,^^3^ Iran is a middle-income country according to the World Bank classification.^4^ Recent reports have shown that the prevalence of stroke in Iran is significantly higher than that in western countries; this is especially true for stroke in the young population.^5^^,^^6^ These reports have emerged from northern and central provinces of Iran. In southern Iran, however, information on stroke epidemiology is limited. 

Fars Province is located in southwestern Iran, and Shiraz is its provincial capital. According to a census in 2006, Fars Province had a population of 4.3 million, 60% of them residing in urban areas.^7^ Nemazee Hospital is a tertiary center in Shiraz and admits patients from the entire Fars Province. Ethnic history of Iran abounds with successive waves of occupation and migration, with the largest ethnic group being the Persians. Mitochondrial DNA linage analysis has determined the main lineage to be western Eurasian.^8^ In Iran, life expectancy is about 72 years for women and 69 years for men, which suggests an ageing population perhaps similar to those in developed countries.^9^ Regarding health plans in Iran, about 90% of the Iranians are covered by at least one health insurance carrier. Several types of health organizations are available to provide health coverage and these include social security, medical services, armed forces, private insurances, and charities. The first three organizations cover mainly urban public and private sector employees, as well as members of the armed forces. In 2000, a rural health insurance system was implemented to provide health coverage to rural inhabitants. The main charity provider is “Imam Khomeini Charity Foundation”, which covers individuals with low or no income that is reflective of a low socioeconomic status.^10^ Similar to other regions of Iran, the population of Fars Province is covered by the same health insurance carriers, with those in the low socioeconomic status accounting for approximately 7%. 

This study was performed to provide basic epidemiological data on stroke. Such information has been very scarce in our region. We sought to determine the main epidemiological characteristics of patients with stroke during the last decade in southern Iran and assess the mortality rate associated with all types of stroke in Fars Province.

## Patients and Methods

All patients with any types of stroke (hemorrhagic or ischemic) were admitted to Nemazee Hospital, a major tertiary center affiliated with Shiraz University of Medical Sciences. We considered the International Classification of Diseases, 9th edition-Clinical Modification (ICD-9-CM) and ICD-10-CM codes as recorded in the hospital database. The final diagnosis was determined by a qualified neurologists and then coded by experienced medical record technicians. Over a decade (from March 2001 to September 2011), patients with any stroke were identified from the hospital database using the ICD-9-CM codes for the years 2001 to 2003 and ICD-10-CM for the years 2004 to 2010. The ICD-9 codes included in our cohort are subarachnoid hemorrhage (430), intracerebral hemorrhage (431), unspecified intracranial hemorrhage (432), transient cerebral ischemia (435), acute ill-defined cerebrovascular disease (436), and other ill-defined cerebrovascular disease (437). The ICD-10 codes included in the cohort are subarachnoid hemorrhage (I60), intracerebral hemorrhage (I61), other non-traumatic intracranial hemorrhage (I62), cerebral infarction (I63) and stroke (I64), other cerebrovascular diseases (I67), cerebrovascular disorders in diseases classified elsewhere (I68), and sequel of cerebrovascular disease (I69). The diagnosis of stroke in all patients was based on clinical findings with computed tomography or magnetic resonance imaging, and was confirmed by an experienced neurologist. Patients with epilepsy, brain tumors, cerebral infections, trauma or deficits due to metabolic causes, or incomplete records were excluded. The follow-up time was equal to the duration of hospital stay. Age, sex, area of residence, socioeconomic status, and length of hospital stay were sought for each patient in a specially-designed data matrix. Because there is a lack of a structured rehabilitation system in southern Iran and most patients are discharged regardless of their stroke severity, discharge destination was not assessed in this analysis. This study was conducted and approved by the Ethics Committee of Shiraz University of Medical Sciences (HP29-90). Since the information was gathered from hospital database and included subject identifiers, we requested and obtained and institutional review board waiver of informed consent. 

*Statistical Analysis*

For univariate analysis, Student’s *t* test or Chi-square test was used to compare the mean and proportions of the continuous and categorical variables. A multivariate logistic regression analysis was built for the outcome of hospital mortality with the following covariates: age groups, gender, area of residence, and socioeconomic status. To assess mortality with the basic demographics of age and gender, a stratified analysis by decades was performed. The trend of mortality over the first and last years in the study period was assessed using the chi-squared test. A probability value less than 0.05 was considered significant. Statistical package for social sciences (SPSS version 15.0) was used for all the statistical tests. 

## Results

*Cohort Demographics*

Medical records of 16351 patients, consisting of 8759 (53.6%) men and 7592 (46.4%) women, were reviewed. There were 428 (2.6%) patients in the pediatric age group (age<18), 2326 (14.2%) young adults (age=19-45), 5958 (36.4%) middle-aged individuals (age=46-64), and 7639 (46.7%) older adults (age>65). The mean age for the entire sample was 63.4 (95% CI: 63.1 to 63.6). Females were slightly older than males (63.8 [95% CI: 63.4 to 64.1] vs. 63.0 years [95% CI: 62.6 to 63.4]; P<0.001). Eighteen percent (n=2935) of our cohort resided in rural areas and the rest lived in urban areas. Patients from rural areas were significantly younger (59.7 [95% CI: 59.0 to 60.5] vs. 63.5 [95% CI: 63.2 to 63.9]; P<0.001). The mean hospital stay was 6.3 days (95% CI: 6.2 to 6.4), which was longer in the pediatric age group than in the adult population (9.4 [95% CI: 8.6 to 10.3] vs. 6.2 [95% CI: 8.6 to 10.3]; P=0.001). Table 1 shows the age distribution of the stroke patients in comparison to the age distribution of Fars Province in 2006, when the national census was performed.

**Table 1:** Age distribution of the stroke patients admitted to Nemazee Hospital in comparison to that of Fars province in 2006

	**Male**		**Female**		**Total**	
	**Stroke admitted to Nemazee Hospital**	**Fars Population **	**Stroke admitted to Nemazee Hospital**	**Fars Population **	**Patients with stroke admitted to Nemazee Hospital**	**Fars Population **
0-9	21 (3.03)	323375 (14.67)	7 (0.96)	306833 (14.39)	28 (1.96)	630208 (14.53)
10-19	12 (1.73)	499512 (22.66)	6 (0.82)	478563 (22.45)	18 (1.26)	978075 (22.55)
20-29	26 (3.75)	523008 (23.72)	14 (1.91)	518186 (24.30)	40 (2.81)	1041194 (24.01)
30-39	29 (4.18)	317169 (14.39)	30 (4.10)	307960 (14.44)	59 (4.14)	625129 (14.41)
40-49	56 (8.07)	239447 (10.86)	66 (9.02)	227454 (10.67)	122 (8.56)	466901 (10.77)
50-59	94 (13.54)	144314 (6.55)	131 (17.90)	145946 (6.85)	225 (15.78)	290260 (6.69)
60-69	122 (17.58)	75225 (3.41)	129 (17.62)	74736 (3.51)	251 (17.60)	149961 (3.46)
70-79	211 (30.40)	61688 (2.80)	209 (28.55)	51961 (2.44)	420 (29.45)	113649 (2.62)
80-89	113 (16.28)	19319 (0.88)	131 (17.90)	17848 (0.84)	244 (17.11)	37167 (0.86)
90 and more	10 (1.44)	1795 (0.08)	9 (1.23)	2539 (0.12)	19 (1.33)	4334 (0.10)
Total	694	2204852	732	2132026	1426	4336878

*Outcome of Hospital Mortality*

**Figure 1:**
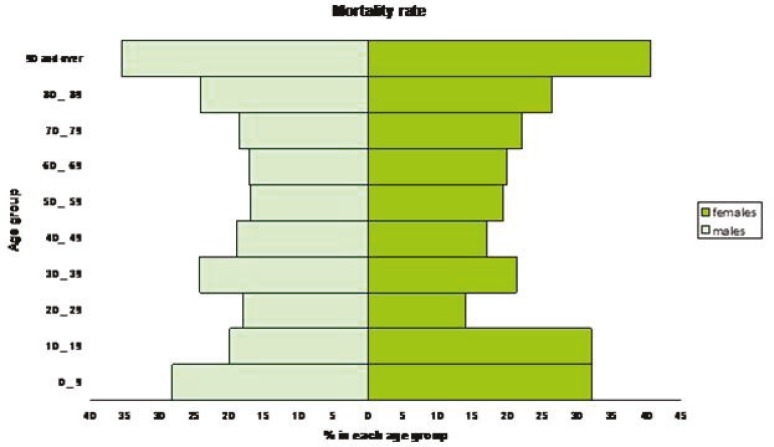
Stratified analysis of age and sex associated with hospital mortality in patients with all types of stroke in Southern Iran

A total of 3354 (20.5%) patients (95% CI: 20.2% to 20.8%) died during the same hospitalization. Table 2 illustrates the basic demographics of those who died compared to the rest of the sample. Those who died were older (mean age=64.3 [95% CI: 63.7 to 65.0] vs. 63.1 [95% CI:62.8 to 63.4]; P<0.001) and they were of a lower socioeconomic status (23.3% [95% CI: 21.8% to 24.8%] vs. 20.4% [95% CI: 20.0% to 20.7%]; P=0.044). Despite the slight male predominence among those who died (50.8% vs. 49.2%), univariate analysis showed a higher mortality among women (male mortality=19.5% [95% CI: 19.1% to 19.9%] vs. female mortality=21.7% [95% CI: 21.2% to 22.2%]; P<0.001). Stratified analysis for age and sex in association with hospital mortality is depicted in figure 1. The mean hospital stay in the patients who died during the same hospitalization was longer than that of the surviving population (7.0 [95% CI: 6.7 to 7.25 days] vs. 6.1 [95% CI: 6.0 to 6.24 days]; P<0.001). Covariates associated with higher hospital mortality in multiple logistic regression analysis were sex (females vs. males OR: 1.15, 95% CI: 1.07 to 1.24; P<0.001), stroke in children compared to those older than 18 (OR: 1.54, 95% CI: 1.24 to 1.91; P<0.001), low socioeconomic status (OR: 1.03, 95% CI: 0.92 to 1.15; P=0.618), and geographic location (rural vs. urban OR: 1.17, 95% CI: 0.99 to 1.39; P=0.065) (table 3). Univariate logistic regression analysis failed to show any difference in terms of mortality between the young adults and the older age groups (OR: 1.05, 95% CI: 0.94 to 1.17; P=0.409). 

**Table 2:** Univariate demographic analysis of stroke mortality in Southern Iran

**Covariate**		**Discharged** **(n=12997)**	**Died** **(n=3354)**	**P value**
Age, mean±SD		63.1±17.2	64.3±18.8	<0.001
Age	≤18	309 (2.4%)	119 (3.6%)	<0.001
	19-45	1525 (11.7%)	373 (11.1%)	
	46-64	3918 (30.1%)	849 (25.3%)	
	≥65	7245 (55.8%)	2013 (60.0%)	
Gender	Female	5943 (45.7%)	1649 (49.2%)	<0.001
	Male	7054 (54.3%)	1705 (50.8%)	
Length of stay (days, mean±SD)		6.13±6.324	6.99±7.661	<0.001
Socioeconomic status	Low	626 (5.1%)	190 (5.6%)	0.044
	Others	12371(94.9%)	3164(94.4%)	
Residency	Rural areas	1783 (13.7%)	449 (13.4%)	0.618
	Urban areas	11214 (86.3%)	2905 (86.6%)	

**Table 3:** Covariates associated with hospital mortality based on multiple logistic regression analysis

		**Odds ratio **	**95.0% CI for Odds ratio**		**Sig.**
			**Lower**	**Upper**	
Sex	Male	1	-	-	<0.001
	Female	1.15	1.07	1.24	
Age	More than 18 year	1	-	-	<0.001
	Under 18 years	1.54	1.24	1.91	
Socioeconomic	Not low	1	-	-	0.618
	Low	1.03	0.92	1.15	
Geographic location	Urban	1	-	-	0.065
	Rural	1.17	0.99	1.39	

*Trends of Mortality Over Time*

**Figure 2:**
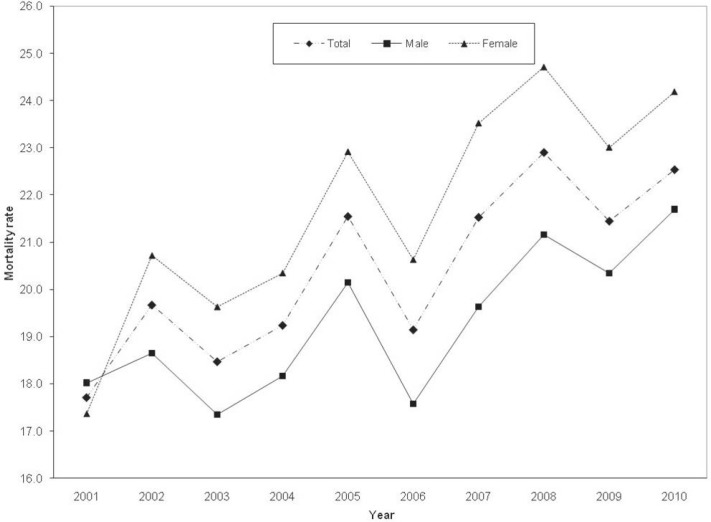
Trend of overall mortality associated with all types of stroke in southern Iran between 2001 and 2010

During the study period, the percentage of all types of stroke admissions to Nemazee Hospital decreased from 5% (95% CI: 4.9% to 5.1%) in 2001 to 4.5% (95% CI: 4.4% to 4.6%) in 2010 (P<0.001). However, the mortality rate among the hospitalized stroke patients (figure 2) increased from 17.7% (95% CI: 16.7% to 18.7%) to 22.2% (95% CI: 21.6% to 23.4%) (P<0.001). This observation was made in both genders. 

## Discussion

Four important observations can be made from this analysis. First is the higher in-hospital mortality (20%) in comparison to developed countries.^1^ Our result chimes in with the reported case fatality rate from any stroke in central Iran (24.6%).^6^ Furthermore, mortality rates in central and southern Iran are higher than those reported from the nearby states. Thirty-day case fatality rate for stroke in Arab middle-eastern and North African countries, where socioeconomic characteristics of the population are generally similar to Iran, falls between 10% and 17.3%.^11^ Several factors may have contributed to these results, including absence of health institution infrastructure such as specialized stroke units and underutilization of thrombolysis, both of which are known to positively influence outcomes in acute ischemic stroke.^12^ Moreover, stroke awareness is lacking among most of the Iranian general population.^13^ This can lead to the referral of stroke patients in late stages and increased mortality. Post-stroke care has been another issue which may have influenced outcome. Surveys of Iranian stroke survivors suggested that the social, financial, and rehabilitative support for stroke was inadequate.^14^ Unlike developed countries, nursing facilities are not available in Iran; consequently, most stroke survivors are discharged home.^6^ The lack of organized rehabilitation care and the nonsystematic nature of family care can lead to lengthy recovery, probable readmissions, and perhaps higher mortality.^15^

The second observation from this analysis is noted differences in epidemiological characteristics of the stroke population in Iran. Our results suggest that a higher proportion of stroke occurs in young adults and children (14% of all stroke cases occurred in those younger than 45). These rates are comparable to those reported in the nearby countries such as Qatar (18%) and Libya (19.1%),^16^^,^^17^ but they are certainly higher than those reported in the western countries.^18^ The in-hospital mortality rate for this group was 21.2%, which was higher than the 3.4% to 11.2% 30-day case fatality rate in Norwegian^19^ and Italian^20^ patients with young-adult stroke. This suggests that stroke afflicts a large number of patients in their reproductive years in Iran, with higher-than-expected mortality.

Thirdly, multiple logistic regression revealed significant higher in-hospital mortality in women and children but not in patients with low socioeconomic status or from rural areas. Similar to prior reports, we observed a slight male predominence in our sample; however, the mortality was higher in women compared to men. The high incidence of stroke mortality in women is probably due to longer life expectancy.^21^ Poor prognosis of stroke in the pediatric age group can be explained by devastating underlying general causes which make the final outcome poor.^22^

The last observation is the disturbing trend of a higher mortality rate over the study period (between 2001 and 2010). This stands in contrast to the recent trends reported from developed and a few developing countries.^23^ The exact explanations are yet to be determined; nevertheless, contributing factors similar to those highlighted above may have played a role in this trend. 

Some shortcomings in this study are worth mentioning. First, this study is a retrospective single-hospital experience and might as such not be reflective of national Iranian standards. Second, the relatively high in-hospital mortality rate should be interpreted with caution because patients with a worse prognosis may have been over-represented among the patients who were admitted to our tertiary referral center. Third, our cohort was identified based on the ICD-9 and ICD-10 coding systems; thus, coding error could not be eliminated. Fourth, stroke-specific characteristics such as stroke location, stroke severity scale, and 30-day mortality were not reported. Fifth, the specific causes of death were not determined according to the hospital database characteristics.

## Conclusion

Our study reconfirmed that stroke is a crucial health problem in Iran. In comparison to western countries, a larger proportion of Iranian patients were young adults and the mortality rate was higher. Although Iran is considered a middle-income country, the allocation of resources to improve the health system may need to be revisited. There is an urgent need for Iranian hospitals to develop better measures to manage acute stroke patients. In a wider context, international organizations should propose guidelines to implement a specialized infrastructure for stroke care in developing countries; these guidelines may influence global outcomes associated with stroke.
